# Tick-host conflict: immunoglobulin E antibodies to tick proteins in patients with anaphylaxis to tick bite

**DOI:** 10.18632/oncotarget.15243

**Published:** 2017-02-09

**Authors:** Lourdes Mateos-Hernández, Margarita Villar, Angel Moral, Carmen GarcíaRodríguez, Teresa Alfaya Arias, Verónica de la Osa, Francisco Feo Brito, Isabel G. Fernández de Mera, Pilar Alberdi, Francisco Ruiz-Fons, Alejandro Cabezas-Cruz, Agustín Estrada-Peña, José de la Fuente

**Affiliations:** ^1^ SaBio, Instituto de Investigación de Recursos Cinegéticos, IREC-CSIC-UCLM-JCCM, Ciudad Real, Spain; ^2^ Department of Allergy, Hospital Virgen del Valle, Toledo, Spain; ^3^ Allergy Section, General University Hospital of Ciudad Real, Ciudad Real, Spain; ^4^ Institute of Parasitology, Biology Center of the Academy of Sciences of The Czech Republic, České Budějovice, Czech Republic; ^5^ Faculty of Science, University of South Bohemia, České Budějovice, Czech Republic; ^6^ Department of Animal Pathology, Faculty of Veterinary Medicine, University of Zaragoza, Miguel Servet, Zaragoza, Spain; ^7^ Department of Veterinary Pathobiology, Center for Veterinary Health Sciences, Oklahoma State University, Stillwater, Oklahoma, USA

**Keywords:** immunology, allergy, alpha-Gal, anaphylaxis, proteomics, Immunology and Microbiology Section, Immune response, Immunity

## Abstract

Tick-borne infectious diseases and allergies are a growing problem worldwide. Tick bite allergy has been associated with the direct effect of immunoglobulin E (IgE) response to tick salivary antigens, or secondary to the induction of allergy to red meat consumption through IgE antibodies against the carbohydrate α-Gal (Gal α 1-3Gal β 1-(3)4GlcNAc-R). However, despite the growing burden of this pathology, the proteins associated with anaphylaxis to tick bite have not been characterized. To address this question, a comparative proteomics approach was used to characterize tick proteins producing an IgE antibody response in a healthy individual with record of tick bites, which had not resulted in any allergic reactions, and two patients with anaphylactic reactions to *Rhipicephalus bursa* or *Hyalomma marginatum* tick bites. Both patients and the healthy individual were red meat tolerant. The results supported a patient-specific IgE antibody response to tick species responsible for the anaphylaxis to tick bite. Both patients and the healthy individual serologically recognized tick proteins with and without α-Gal modifications, with proteins differentially recognized by patients but not control sera. These proteins could be used as potential antigens for diagnostics, treatment and prevention of tick bite-induced allergies.

## INTRODUCTION

Ticks are blood-sucking ectoparasites that feed on different vertebrate hosts to complete their life cycle [1]. Humans are accidental hosts, but tick-borne diseases are a growing problem worldwide [2, 3]. Tick modulate host immunity through salivary gland proteins that are injected into the host during blood feeding to suppress inflammatory responses and facilitate feeding and pathogen transmission [4, 5].

The first case of anaphylaxis, secondary to allergy to tick bite was reported in Australia in 1940 [6]. Although this problem remains most prominent in Australia, where fatality cases have been recorded [7], anaphylaxis to tick bite has been also reported in the United States and Europe [8, 9]. The morbidity associated with tick bite allergy has been associated with the direct effect of immunoglobulin E (IgE) response to tick salivary antigens [7–10], or secondary to the induction of allergy to red meat consumption through IgE antibodies against the carbohydrate α-Gal (Gal α 1-3Gal β 1-(3)4GlcNAc-R) [11–20]. Humans do not synthesize α-Gal and healthy individuals develop a potent immune response against this carbohydrate widely present on tissues of nonprimate mammals [21, 22]. Recently, van Nunen et al. [17] reported that patients living in a tick endemic region of Australia developed red meat allergy after experiencing large local reactions to tick bites. This finding led to the suggestion that tick-induced allergies to red meat and other compounds may occur after anaphylactic reactions to tick bite [23].

The anaphylaxis to tick bite has been associated with a variety of both soft- and hard-bodied tick species [8–10, 24]. However, despite the growing burden of this pathology worldwide [7], the proteins associated with anaphylaxis to tick bite have not been characterized. To address this question, the goal of this study was to use a comparative proteomics approach to characterize tick proteins producing an antibody response in a healthy individual with record of tick bites, which had not resulted in any allergic reactions, and two patients with anaphylactic reactions to *Rhipicephalus bursa* or *Hyalomma marginatum* tick bites. Both patients and the healthy individual were red meat tolerant. The results suggested proteins that could be used as potential antigens for the diagnostics, treatment and prevention of tick bite-induced allergies.

## RESULTS

### Diagnosis of patients with immediate anaphylaxis to tick bite but without delayed anaphylaxis to mammalian red meat

**Figure 1:**
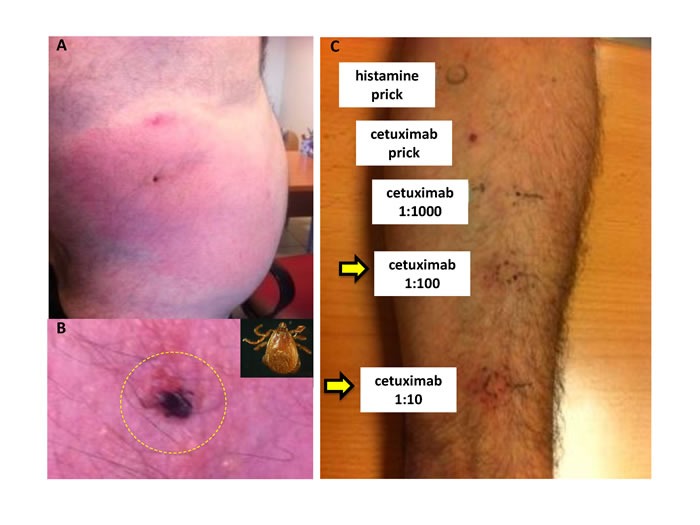
Case presentation for patient 1 **A**. The anaphylactic reaction was diagnosed in patient 1 after *R. bursa* tick bite that resulted in generalized itching, difficult breathing, nausea and somnolence that required medical attention. **B**. Female *R. bursa* attached to patient's skin and shown in more detail in the inset. **C**. Patient´s positive intradermal reaction to cetuximab (1:100 to 1:10 dilution; arrows). Intradermal reaction to cetuximab (1:1000 dilution) and to cetuximab prick were negative. Histamine prick was used as positive skin test control.

**Figure 2:**
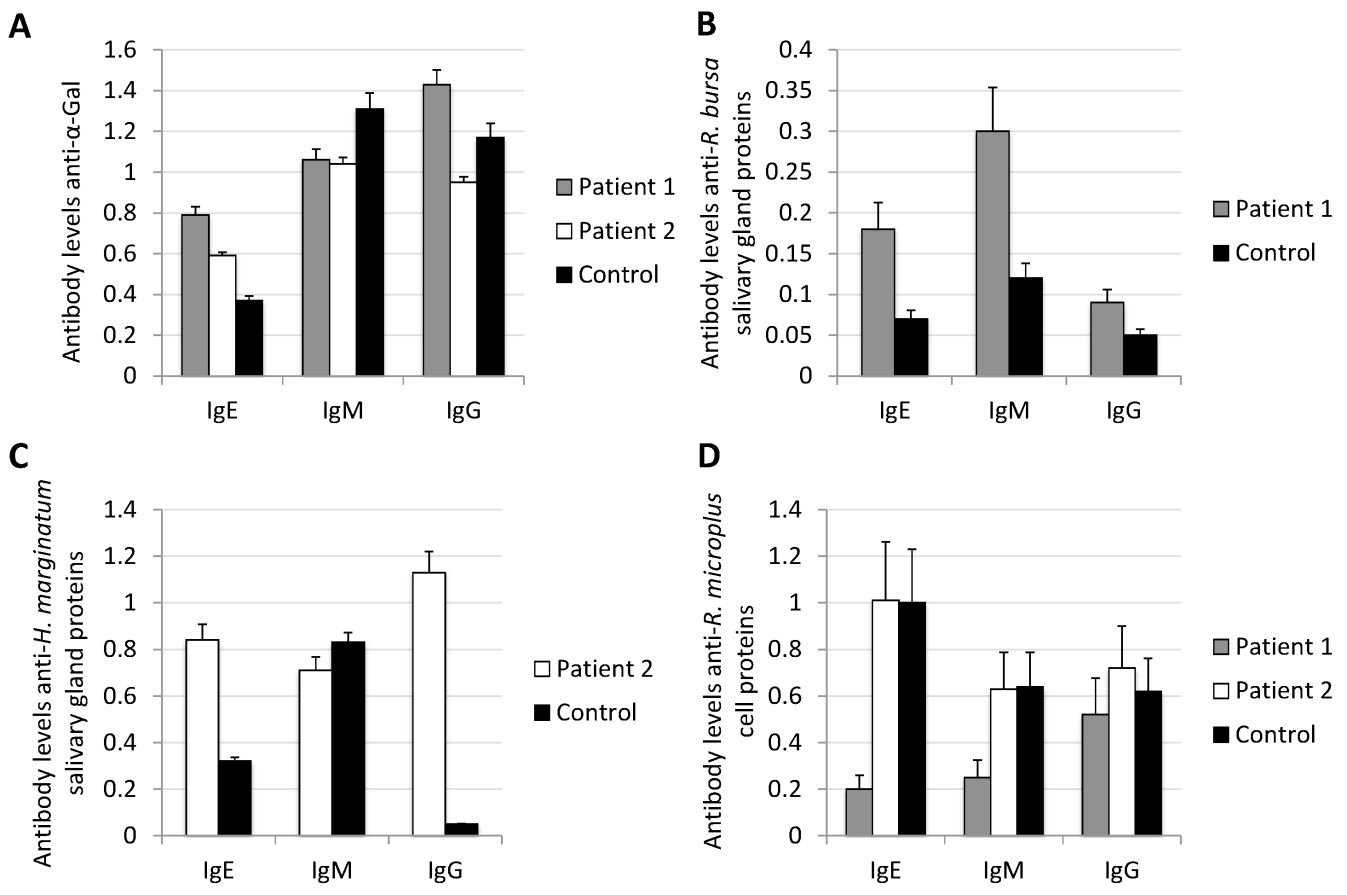
Immunological response to tick proteins The IgE, IgM and IgG antibody levels were determined by ELISA in patients and control serum samples against **A**. α-Gal, **B**. *R. bursa* salivary gland proteins, **C**. *H. marginatum* salivary gland proteins, and **D**. *R. microplus* BME/CTVM23 tick cell proteins. Antibody levels were determined as OD at 450 nm and shown as average + SD of 4 technical replicates.

Two unrelated adult male patients diagnosed with immediate anaphylaxis to *R. bursa* (patient 1; Figures 1A and 1B) or *H. marginatum* (patient 2) tick bite, but without delayed anaphylaxis to red meat consumption were selected for the study. An unrelated adult male with record of tick bites, which had not resulted in any allergic reactions, was also included in the study as a healthy control individual. Skin prick tests to commercial food allergens from pork, horse, lamb, rabbit, ostrich meats, beef and bovine serum albumin (BSA) were negative in both patients, in correspondence with the absence of reports of allergic reactions to read meat consumption. The IgE levels to commercial allergens including pork, lamb, rabbit and BSA were also negative ( < 0.35 kU/l). However, patient 1 but not patient 2 had a positive intradermal reaction to cetuximab (1:100 to 1:10 dilution; Figure 1C). The total IgE content and response to α-Gal determined using the ImmunoCAP Phadia kits were higher in patient 1 (128.0 kU/l and 3.5 kU/l, respectively) than in patient 2 (51.2 kU/l and 0.01 kU/l) and healthy control individual (23.3 kU/l and 0.02 kU/l). Furthermore, the anti-α-Gal IgE and IgG antibody titers determined by ELISA were also higher in patient 1 than in patient 2 and the healthy individual (Figure 2A).

The analysis of serum samples identified IgE, IgM and IgG antibodies against *R. bursa* and *H. marginatum* salivary gland, and *Rhipicephalus microplus* BME/CTVM23 cell proteins in both patients and control individual (Figures 2B-2D). In general, the antibody levels against tick salivary gland proteins were higher in patients than in control individual (Figures 2B and 2C). However, the antibody levels against *R. microplus* cell proteins tend to be lower in patient 1 than in patient 2 and healthy control (Figure 2D).

**Figure 3:**
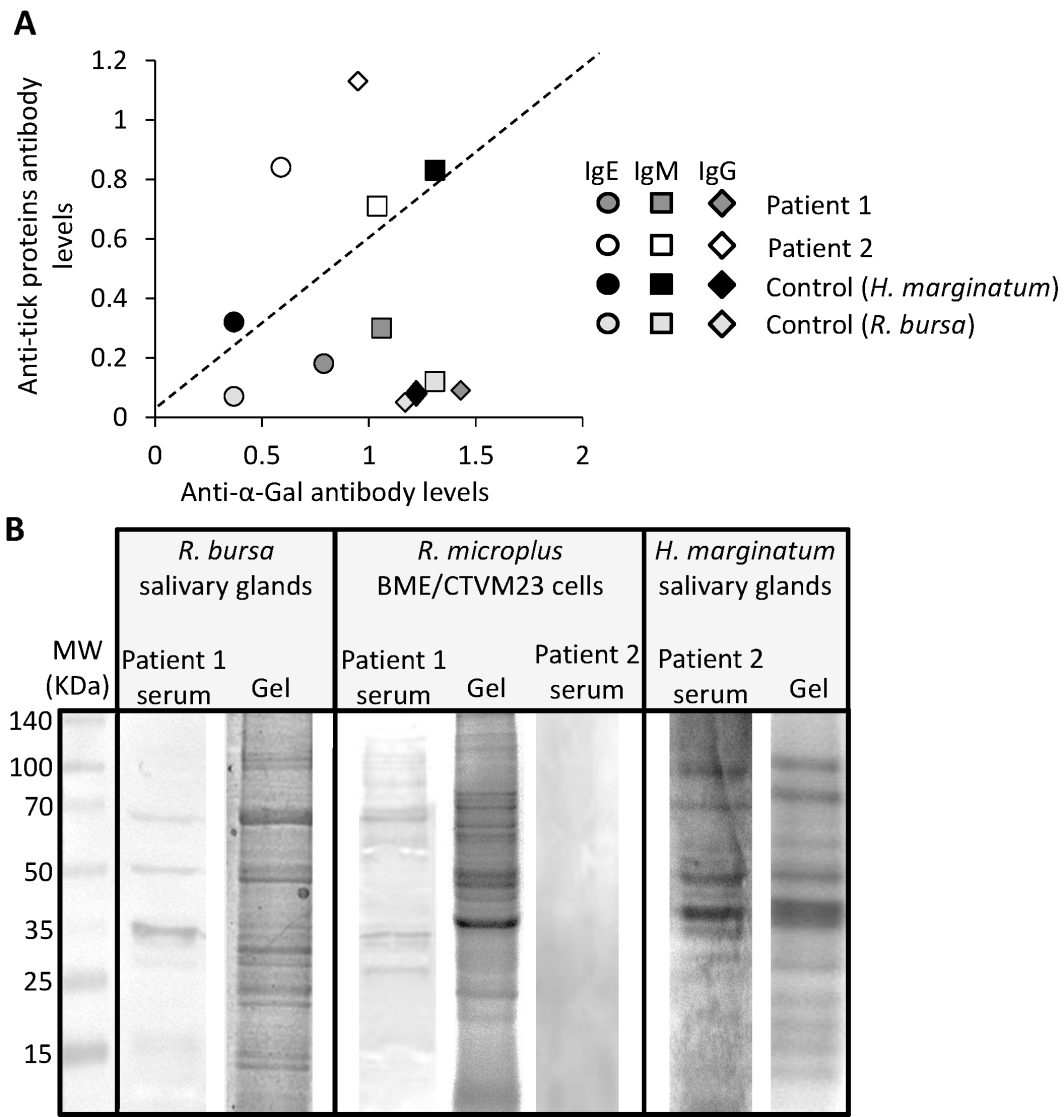
Patient-specific antibody response to tick species responsible for the reported anaphylactic reaction to tick bite **A**. Correlation analysis between IgE, IgM and IgG antibody levels against *R. bursa* or *H. marginatum* tick proteins and α-Gal in patients 1 and 2 and healthy control individual. Antibody levels were determined as OD at 450 nm and shown as the average of 4 technical replicates. **B**. The IgE response to *R. bursa* and *H. marginatum* salivary gland and *R. microplus* BME/CTVM23 tick cell proteins was analyzed by 1-D Western blot using patient 1 and 2 sera. Abbreviation: MW, molecular weight protein marker.

These results supported the diagnosis of anaphylaxis to tick bite in patients 1 and 2, and suggested the possible implication of anti-α-Gal IgE response in patient 1 but not in patient 2. However, the immediate anaphylaxis after tick bite in patients 1 and 2 appeared not to be related to red meat consumption, and both patients continue consuming red meat without any allergic reactions. Furthermore, no correlation was found between IgE, IgM or IgG antibody levels to tick proteins and α-Gal in both patients and the healthy individual (Figure 3A). The antibody response was characterized using similar methods, therefore suggesting that the absence of correlation between the antibody levels to tick proteins and α-Gal reflects a differential response to these antigens.

### Identification and characterization of tick proteins recognized by patient and healthy individual control sera and reactive with anti-α-Gal antibodies

**Figure 4:**
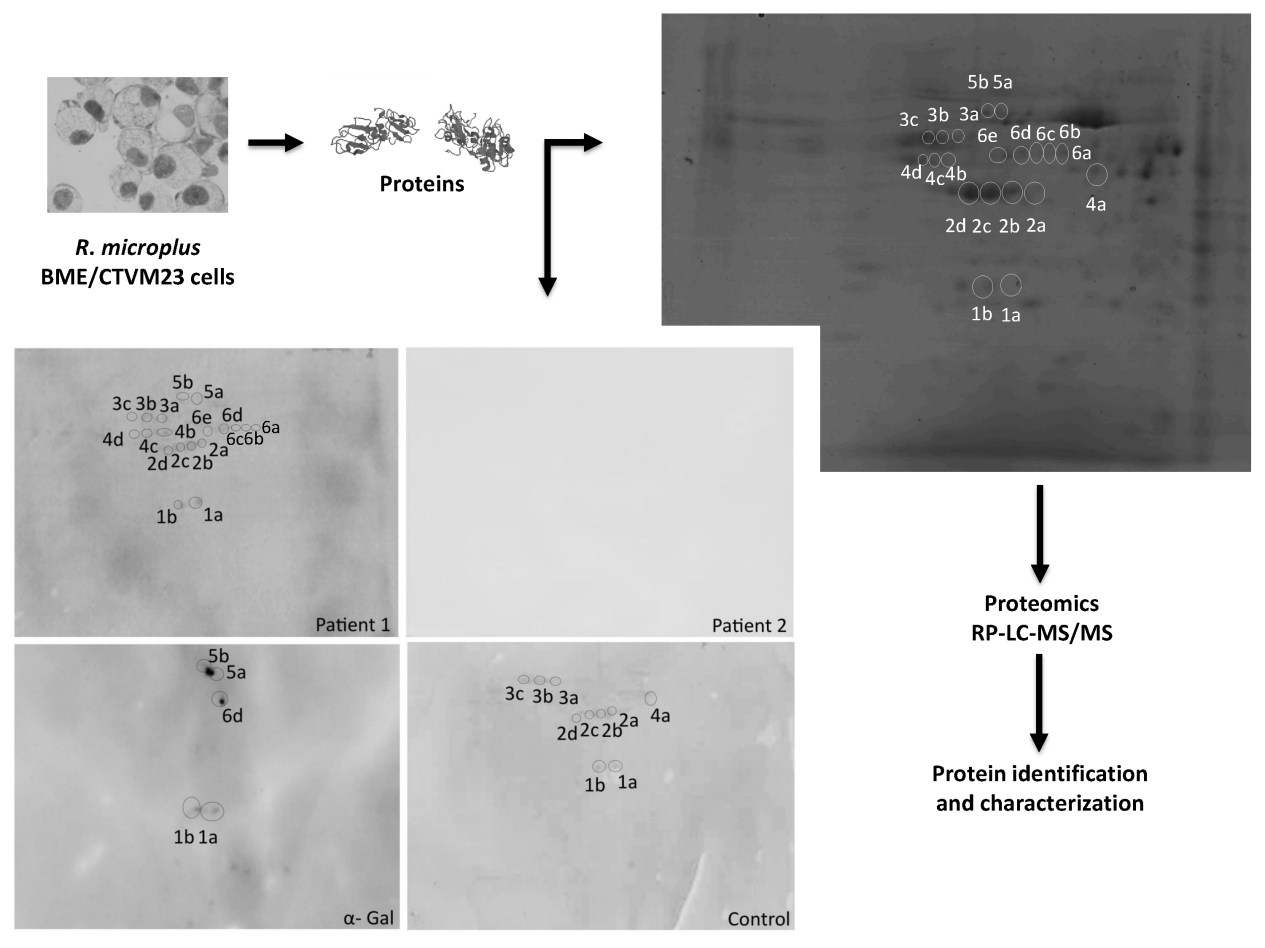
***Rhipicephalus*** tick proteins recognized by IgE in patient 1 and control sera and by anti-α-Gal IgE antibodies. The *R. microplus* BME/CTVM23 tick cell proteins were extracted and analyzed by 2-D Western blot using patients and control sera and anti-α-Gal antibodies. The protein spots of interest recognized by patients or control sera and by anti-α-Gal antibodies were manually excised from the stained gel and used for proteomics analysis. The same settings were used for all four panels in which proteins were resolved by isoelectrical focusing at pH 3-11 followed by 12% SDS gel electrophoresis in the second dimension with 140-15 kDa molecular weight range.

**Figure 5:**
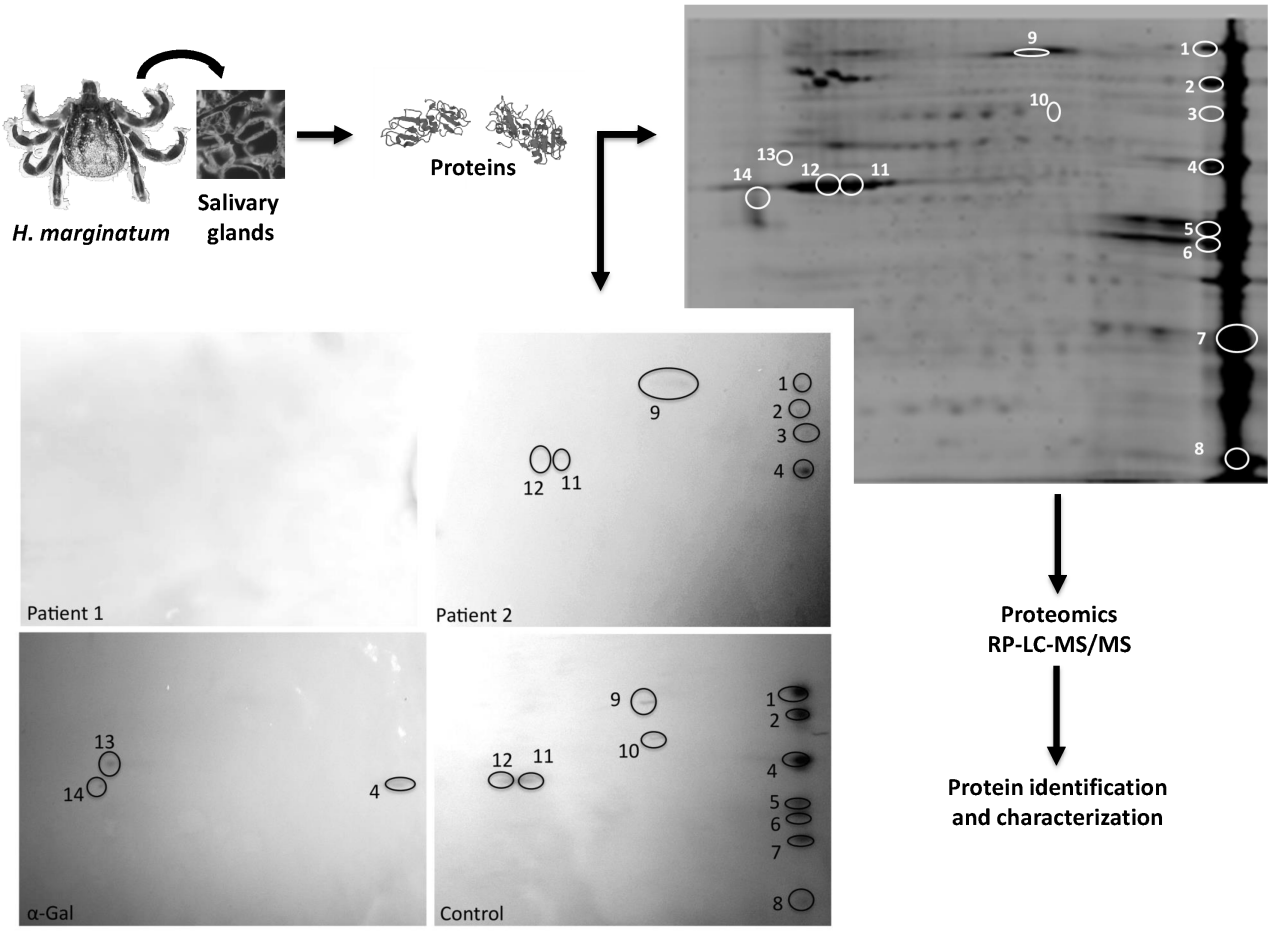
***Hyalomma*** tick proteins recognized by IgE in patient 2 and control sera and by anti-α-Gal IgE antibodies. The *H. marginatum* salivary glands were dissected and proteins were extracted and analyzed by 2-D Western blot using patients control sera and anti-α-Gal antibodies. The protein spots of interest recognized by patient or control sera and by anti-α-Gal antibodies were manually excised from the stained gel and used for proteomics analysis. The same settings were used for all four panels in which proteins were resolved by isoelectrical focusing at pH 3-11 followed by 12% SDS gel electrophoresis in the second dimension with 140-15 kDa molecular weight range.

To identify tick proteins that react with patient and control sera, and potentially containing α-Gal, *R. bursa* and *H. marginatum* salivary gland and *R. microplus* BME/CTVM23 tick cell proteins were analyzed by one-dimensional (1-D) and two-dimensional (2-D) Western blot using patient and control sera, and anti-α-Gal antibodies (Figures 3B-5).

First, a 1-D Western blot was used to characterize the IgE antibody response to tick proteins in patients 1 and 2 (Figure 3B). The results showed a protein recognition pattern with patient 1 serum against *R. bursa* salivary glands and *R. microplus* tick cell proteins (Figure 3B). However, patient 2 serum reacted against *H. marginatum* salivary gland proteins but not against *R. microplus* tick cell proteins (Figure 3B). These results agreed with the ELISA results showing that both patients have antibodies against tick proteins (Figures 2B and 2C), but suggested a patient-specific antibody response to tick species responsible for the reported anaphylactic reaction to tick bite (i.e. *Rhipicephalus* spp. in patient 1 and *H. marginatum* in patient 2).

**Figure 6:**
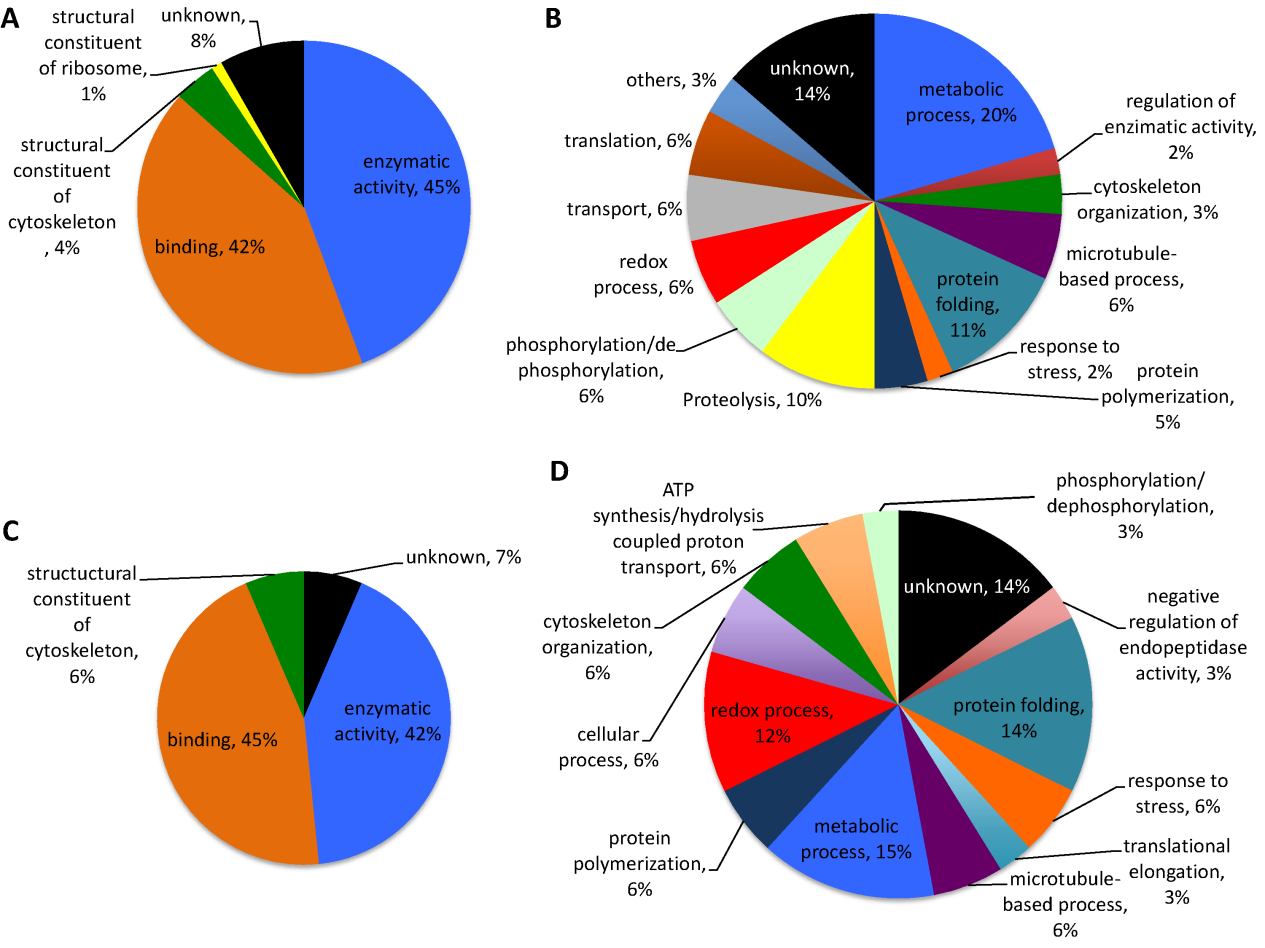
Gene ontology for ***Rhipicephalus*** tick proteins recognized by IgE in patient 1 and control sera and by anti-α-Gal IgE antibodies. The *R. microplus* BME/CTVM23 tick cell proteins identified by proteomics analysis were functionally annotated for molecular function and biological process. **A**. Molecular function for unique proteins identified by patient and control sera. **B**. Biological process for unique proteins identified by patient and control sera. **C**. Molecular function for proteins recognized by anti-α-Gal antibodies. **D**. Biological process for proteins recognized by anti-α-Gal antibodies.

The *R. microplus* BME/CTVM23 tick cells and *H. marginatum* salivary gland proteins were then used for higher resolution 2-D gel Western blots and mass spectrometry (MS) to identify proteins differentially recognized by IgE in patients but not control sera and reacting with anti-α-Gal antibodies (Figures 4 and 5). A total of 139 *R. microplus* BME/CTVM23 tick cell proteins were identified from the 20 spots recognized by patient 1 or control sera and by anti-α-Gal antibodies in the 2-D gels (Figure 4 and Supporting information Data Set 1). As in the 1-D Western blot analysis (Figure 3B), no proteins were recognized by patient 2 serum (Figure 4). Of the identified proteins, 70 were unique (not repeated between protein spots) (Supporting information Data Set 1). These unique proteins had a high representation in enzymatic activity and binding molecular functions, accounting for 87% of all proteins (Figure 6A), and in metabolic process, protein folding and proteolysis biological processes, accounting for 41% of all proteins (Figure 6B). Thirty-one proteins were identified in spots positive for α-Gal (Figure 4 and Supporting information Data Set 1), with similar representation for molecular function (Figure 6C) and biological process (Figure 6D) ontology to all identified proteins (Figures 6A and 6B). Of the 70 identified unique proteins, only 29 had cellular component assignments with a prevalent localization in the cytoplasm (14/29; 48%) (Supporting information Data Set 1). However, of the 70 unique proteins identified in *R. microplus* BME/CTVM23 tick cells, 65 (93%) had orthologs in the sialome reported in other tick species (Supporting information Data Set 1), suggesting that these proteins may be present in tick saliva.

**Figure 7:**
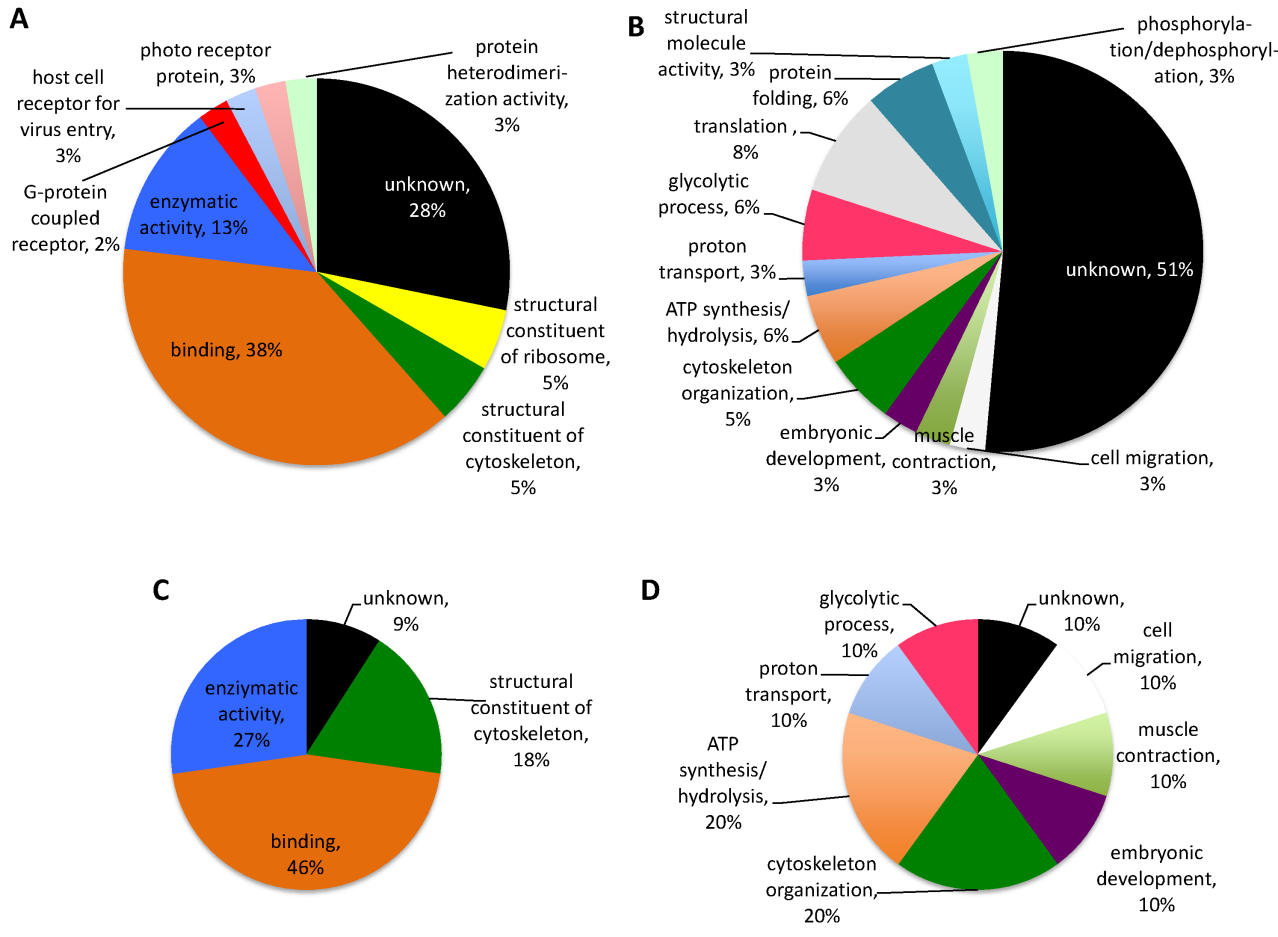
Gene ontology for ***Hyalomma*** tick proteins recognized by IgE in patient 2 and control sera and by anti-α-Gal IgE antibodies. The *H. marginatum* salivary gland proteins identified by proteomics analysis were functionally annotated for molecular function and biological process. **A**. Molecular function for unique proteins identified by patient and control sera. **B**. Biological process for unique proteins identified by patient and control sera. **C**. Molecular function for proteins recognized by anti-α-Gal antibodies. **D**. Biological process for proteins recognized by anti-α-Gal antibodies.

In *H. marginatum* salivary gland proteins, a total of 32 proteins were identified from the 14 spots recognized by patient 2 or control sera and by anti-α-Gal antibodies in the 2-D gels (Figure 5 and Supporting information Data Set 1). Supporting the patient-specific antibody response to tick species responsible for the reported anaphylactic reaction to tick bite, patient 1 serum did not recognize *H. marginatum* proteins (Figure 5). Of the identified proteins, 28 were unique (not repeated between protein spots) (Supporting information Data Set 1). As in *R. microplus* cell proteins, enzymatic activity and binding were the most represented molecular functions, accounting for 51% of all proteins (Figure 7A). However, for biological process ontology differences were observed between *R. microplus* cells and *H. marginatum* salivary gland proteins, in which 51% of the proteins could not be assigned to a biological process (Figure 7B). For example, although protein folding was present in both datasets (Figures 6B and 7B), translation and glycolytic processes were better represented in *H. marginatum* than in *R. microplus* proteins. Nevertheless, protein processing in general was highly represented in both *R. microplus* and *H. marginatum* identified unique proteins (Figures 6B and 7B). Six proteins were identified in spots positive for α-Gal (Figure 5 and Supporting information Data Set 1), and as in *R. microplus* proteins (Figures 6A and 6C), showed a similar representation for molecular function (Figure 7C) than for all identified unique proteins (Figure 7A). For the biological process ontology of proteins identified in spots positive for α-Gal (Figure 7D), the most represented processes such as cytoskeleton organization, ATP synthesis/hydrolysis, proton transport, glycolytic process, cell migration, muscle contraction, and embryonic development accounting for 90% of all proteins were also represented in all *H. marginatum* (Figure 7B) and *R. microplus* (Figures 6B and 6D) identified unique proteins. As in *R. microplus* cells, most of the 28 unique proteins identified in *H. marginatum* salivary glands with cellular component assignments were cytoplasmic and intracellular (9/12; 75%) (Supporting information Data Set 1). However, as expected for salivary gland proteins, 96% of the 28 unique proteins identified in *H. marginatum* had orthologs in the sialome reported in other tick species (Supporting information Data Set 1), supporting that these proteins are present in tick saliva.

These results supported a patient-specific antibody response to tick species responsible for the reported anaphylactic reaction to tick bite. The tick proteins recognized by patient and control sera showed a high representation in the enzymatic activity and binding molecular functions, and in protein processing biological process. Most of the identified proteins were present in the sialome reported in other tick species, supporting that these proteins are or may be present in tick saliva and therefore secreted to the host during tick feeding. Finally, some of the tick proteins were identified in spots positive for α-Gal, suggesting the presence of this post-translational modification in some of these proteins.

### Identification and characterization of tick proteins differentially recognized by patient but not healthy individual control sera

Tick proteins that react with IgE in patients but not control sera were identified and characterized as a potential source for the development of anaphylactic reactions to tick bite. In *R. microplus* tick cells, 61 proteins (35 unique) were recognized by patient 1 serum only, of which 31 proteins (21 unique) were positive for α-Gal (Table 1 and Supporting information Data Set 1). Five unique proteins were recognized by control serum only, while 73 proteins (38 unique) were positive against both patient 1 and control sera (Supporting information Data Set 1). In *H. marginatum* salivary glands, only two unique proteins were cognized by patient 2 but not control sera and were not positive for α-Gal (Table 1 and Supporting information Data Set 1). Sixteen unique proteins were recognized by control serum, and none of them were positive for α-Gal (Supporting information Data Set 1). Tick proteins differentially recognized by IgE in patients sera and positive for α-Gal could not be grouped by common molecular function or biological process categories (Table 1), suggesting that the role of these proteins in developing anaphylaxis to tick bite may not be related to protein function.

**Table 1:** Tick proteins differentially recognized by IgE in patients but not healthy individual control sera and reacting or not with anti-α-Gal antibodies

Accession No.	Description	Reactive against serum	
		Patients	Anti-α-Gal
**Patient 1 (*R. microplus*** **BME/CTVM23 cell proteins)**			
A0A034WWU3	Alpha2 macroglobulin 2	+	+
A0A034WXE0	Heat shock protein 70 1	+	+
A0A034WXL0	Heat shock protein 90 1	+	+
A0A034WXY9	Heat shock protein 70 cognate	+	+
A0A034WYY9	Elongation factor 1-alpha	+	-
A0A034WZ70	Alpha tubulin 1	+	+
L7LU17	Putative hydroxyacyl-coenzyme a dehydrogenase/3-ketoacyl-coenzyme a thiolase/enoyl-coenzyme a hydrat	+	+
L7LUC2	Adenylyl cyclase-associated protein	+	+
L7LVV5	Putative klingon	+	+
L7LW52	Isocitrate dehydrogenase [NADP]	+	-
L7LX08	Putative molecular chaperones mortalin/pbp74/grp75 hsp70 superfamily	+	+
L7M2Y0	Putative igf-ii mrna-binding protein imp	+	+
L7M4I4	Putative nucleotide excision repair factor nef2 rad23 component	+	-
L7M612	Putative ubiquitin regulatory protein	+	+
L7M755	Putative nadh-ubiquinone oxidoreductase ndufs1/75 kDa subunit	+	+
L7M782	Putative vacuolar h+-atpase v1 sector subunit b	+	-
L7M817	Putative peptid	+	-
L7M875	Tubulin beta chain	+	+
L7M8B5	Putative spliceome rna helicase ddx39b	+	-
L7M8Z1	Putative dynamitin	+	-
L7MAA0	ATP synthase subunit alpha	+	+
L7MAE4	Putative chaperonin protein	+	+
L7MAG2	Fascin	+	+
L7MAL5	Uncharacterized protein	+	+
L7MAR2	Putative thioredoxin/protein disulfide isomerase	+	+
L7MAS7	Putative tubulin beta 2b class iib	+	-
L7MAT5	Succinyl-CoA ligase subunit beta	+	-
L7MAX7	Putative eukaryotic translation initiation factor 4a2	+	-
L7MBL7	Putative pleckstrin logy domain-containing family f member 2	+	-
L7MD56	Putative neural cell adhesion molecule l1	+	-
L7MDQ8	ATP synthase subunit beta	+	-
L7MEG0	Putative heat shock protein	+	+
L7MHM2	Uncharacterized protein	+	-
L7MIL3	Putative aldehyde dehydrogenase	+	+
L7MJP7	Putative serine/threonine protein kinase gpbp	+	+
Q7YW74	Cathepsin L-like cysteine proteinase B	+	-
**Patient 2 (*H. marginatum*** **salivary gland proteins)**			
A0A131XJ07	Putative alternative splicing factor	+	-
E2J6Q7	Putative cement protein	+	-

## DISCUSSION

The objective of this study was to identify and characterize tick proteins potentially associated with anaphylaxis to tick bite. To address this objective, a comparative proteomics approach was used to characterize the IgE antibody response against tick proteins and α-Gal in a healthy individual and patients with anaphylactic reaction to tick bite but without delayed anaphylaxis to mammalian red meat. Salivary gland proteins from the same tick species to which patients reacted, *R. bursa* and *H. marginatum*, and *R. microplus* BME/CTVM23 tick cell proteins were used for serological, Western blot, and proteomics analyses. Cultured tick cells are models to study tick-host-pathogen interactions [25, 26], and *R. microplus* is the *Rhipicephalus* species with the best genome sequence coverage that facilitates protein assignment after MS analysis [27]. Therefore, protein extracts from the *R. microplus* BME/CTVM23 tick cell line were used for the identification of proteins differentially recognized by patient 1, who developed anaphylaxis to *R. bursa* tick bite.

These results supported a patient-specific IgE antibody response to tick species responsible for the reported anaphylactic reaction to tick bite, and suggested the possible implication of anti-α-Gal IgE response in patient 1, but not in patient 2. Both patients and the healthy individual serologically recognized tick proteins with and without α-Gal modifications. Although protein samples were obtained from different tissues (salivary glands from partially fed female ticks and tick cell culture), which explains differences in protein identification, similar molecular functions and biological processes were identified for the tick proteins recognized by patients and control sera. The tick proteins recognized by patient and control sera are probably involved in tick feeding in humans, which supports a role for the enzymatic activity and binding molecular functions and protein processing biological process in the successful tick feeding on humans [4, 28].

Some of the proteins identified as differentially recognized by patients sera were predicted as localized intracellular. However, orthologs for these proteins were found in the sialome reported in other tick species. Previous reports have shown the presence in the tick sialome of otherwise predicted intracellular proteins, suggesting the activity of non-classical secretion mechanisms such as exosome secretion in tick salivary glands [29]. Therefore, these results probably reflected the IgE antibody response to salivary proteins secreted during tick feeding.

As previously reported in *A. americanum* [12, 19], *I. ricinus* [30] and *H. longicornis* [16], anti-α-Gal IgE antibodies recognized proteins with this modification in *R. bursa* and *H. marginatum* salivary glands, and *R. microplus* BME/CTVM23 tick cells, showing for the first time the presence of α-Gal-modified proteins in *Rhipicephalus* and *Hyalomma* tick species. These results also confirmed previous findings in *H. longicornis* [16], supporting the presence of α-Gal-modified proteins in the sialome of *H. marginatum*.

Recently, we proposed that tick-host-pathogen interactions evolved with conflict and cooperation between parties [31]. The conflict between ticks and hosts includes host local and systemic reactions to tick bite [31]. The comparative proteomics analysis showed the presence of tick proteins that react with IgE in patients but not control sera. The results reported here in *Rhipicephalus* and *H. marginatum* and in other tick species suggested a relationship between tick sialome proteins and the development of anaphylactic reactions to tick bite [7–10, 12, 16, 30]. However, the human immune response to tick bite could have different outcomes [20]. Obviously, many individuals do not develop anaphylactic reactions to tick bite, probably because the immune system is able to produce IgM and IgG responses that have a protective role as opposed to the IgE response [15, 32]. In fact, differences in tick proteins recognized by patients and healthy individual sera may reflect differences in the immune response that affect the development of anaphylaxis. However, in a growing number of cases tick bite results in the development of delayed anaphylaxis to red meat consumption, which has been associated with the IgE response to α-Gal [12–20]. Therefore, tick proteins with putative α-Gal modifications and recognized by patients and control sera may be responsible for sensitization to α-Gal with risk of developing allergy to read meat consumption. At least in some cases, patients with anaphylaxis to tick bite may be at a higher risk of developing allergy to red meat consumption. Finally, as shown here for patient 1, some individuals may react to tick bite with an immediate anaphylaxis that could be also related to anti-α-Gal IgE response, which may increase susceptibility to tick bite [20]. These results also suggested that both immediate anaphylaxis to tick bite and delayed anaphylaxis to red meat consumption might eventually concur in some patients with still unknown differences associated to host immune response, tick species and susceptibility to tick-borne pathogens [15, 20, 23]. Other factors such as tick removal [23] and food-dependent exercise-induced anaphylaxis (FDEIA) [20, 33, 34] may also affect the development of anaphylactic reactions to tick bite. Consequently, additional research is needed to understand the mechanisms by which ticks alter host immune response resulting in immediate anaphylaxis to tick bite and/or delayed anaphylaxis to red meat consumption.

Recently, we proposed that tick proteins reactive with sera of patients with anaphylactic reactions to tick bite and red meat consumption could be used for diagnosis, treatment and prevention of these emerging tick-borne allergies [15]. Tick proteins putatively present in the sialome and reacting with IgE in patients but not control sera could be used for the diagnosis of a predisposing condition for anaphylaxis to tick bite, particularly when highly conserved across different tick species. Furthermore, tick antigens inducing a protective immune response could be used to develop vaccines for the control of tick infestations, transmission of tick-borne pathogens and anaphylaxis to tick bite or red meat consumption [15, 32, 35]. Therefore, sialome proteins with α-Gal modification and recognized by patients but not control sera could be selected as candidate protective antigens for the treatment and prevention of tick-borne anaphylactic reactions and other tick-borne diseases.

## MATERIALS AND METHODS

### Patients and the healthy individual

The use of human peripheral blood serum samples from patients and the healthy individual was done with their informed consent in compliance with the Helsinki Declaration. Nursing personnel at the Hospital Virgen del Valle (Toledo) and the General University Hospital of Ciudad Real, Spain, extracted blood samples.

The first patient (patient 1) was a 55 year-old male with repeated anaphylactic reactions to *R. bursa* tick bites. The patient was working in a goat farm in Toledo Province, Castilla-La Mancha, Spain. About 15 years ago the patient was diagnosed with allergies to cereal flours and leguminous vegetables used for goat feeding and prescribed with corticoids and bronchodilators. Recently, he suffered two episodes of anaphylaxis after *R. bursa* tick bites on different parts of the body that resulted in generalized itching (Figures 1A and 1B), difficult breathing, nausea and somnolence that required medical attention. The patient described the occurrence of previous tick bites without symptoms. Blood samples used in this study were collected after the second anaphylaxis to tick bite.

The second patient (patient 2) was a 52 year-old male without previous record of allergic reactions. He showed generalized itching and erythema, difficult breathing, nausea and somnolence that appeared during his work at a cattle farm in Ciudad Real Province, Castilla-La Mancha, Spain, and required medical attention. This patient was diagnosed with anaphylaxis likely due to *H. marginatum* tick bites [36].

The unrelated healthy control individual was a 55 year-old male with record of tick bites, which had not resulted in any allergic reactions. Both patients and the healthy individual were red meat tolerant.

### Serum sample preparation

For separation of serum from total blood, a sterile tube without anticoagulant was used. The blood from each patient and the healthy individual was maintained in standing position at room temperature (RT) for clotting (20-30 min), and centrifuged at 1,500 x g for 20 min at RT. Serum was collected and conserved at -20 °C until used for analysis.

### Tick collection and protein sample preparation

Unfed *R. bursa* female ticks (*N* = 7) were collected in June 2014 in an area near the goat farm where the patient 1 works (Hontanar, Toledo, Spain; 39.59N-4.50E). Ticks were collected by dragging the vegetation with a 2 × 1 m white cotton flannelette [37], stored refrigerated and quickly transported to the laboratory where they were identified [38], and stored at -20 °C until processed. Partially fed *H. marginatum* female ticks (*N* = 10) were collected at initial stages of feeding from cattle in May 2001 in Son Parc (Menorca, Spain; 40.02N-4.09E), and stored in 70% ethanol until processed. The ticks were then washed with phosphate buffered saline (PBS), dissected and the salivary glands were stored separately in RNAlater (Invitrogen, Carlsbad, CA, USA) and used for protein extraction. For protein extraction, salivary glands were homogenized with a glass homogenizer (20 strokes) in lysis buffer (7M Urea, 2M Thiourea, 2% 3- [(3-cholamidopropyl)dimethylammonio]-1-propanesulfonate, CHAPS) supplemented with complete mini protease inhibitor cocktail (Roche, Basel, Switzerland). Sample was sonicated for 1 min in an ultrasonic cooled bath followed by 10 sec vortex. After 3 cycles of sonication-vortex, the homogenate was centrifuged at 200 x g for 5 min at 4 °C to remove cellular debris. The supernatant was collected and protein concentration was determined using the Bradford Protein Assay (Thermo Scientific, San Jose, CA, USA) with BSA as standard.

### Cultured tick cells and protein sample preparation

The tick cell line BME/CTVM23, derived from *R. microplus* embryos (provided by L. Bell-Sakyi, the Tick Cell Biobank, The Pirbright Institute, UK), was cultured in L15 medium as described previously [25]. Approximately 10^7^ cells were centrifuged at 1,000 x g for 3 min, washed 3 times with PBS and homogenized in lysis buffer following the same protocol described above for the extraction of tick salivary gland proteins.

### Skin tests

Skin prick tests were conducted to a panel of commercially available food allergens (beef, pork, horse, lamb, rabbit, ostrich meats and bovine serum albumin; Bial-Aristegui Laboratory, Bilbao, Spain). Cetuximab, a monoclonal antibody presenting the α-Gal oligosaccharide in the heavy chain and used for the treatment of metastatic colorectal cancer [18, 39], was used in skin tests for diagnosis of α-Gal-induced anaphylaxis [40]. The prick test to cetuximab (Erbitux; Merck SL, Madrid, Spain) was conducted at a concentration of 5 mg/ml and intradermal tests to cetuximab were conducted with 50 μl of 1:10 (0.5 mg/ml), 1:100 (0.05 mg/ml) and 1:1000 (0.005 mg/ml) dilutions as described previously [40]. Histamine prick was used as positive skin test control.

### Determination of anti-allergens IgE antibody titers

The ImmunoCAP-250 analyzer (Phadia, Uppsala, Sweden) was used to determine the IgE levels to commercial allergens (pork, lamb, rabbit, beef and bovine serum albumin) following manufacturer instructions.

### Determination of anti-tick proteins IgG, IgM and IgE antibody titers

The ELISA tests to determine the IgG, IgE and IgM content in response to tick proteins in patients and control sera were performed using *R. bursa* and *H. marginatum* salivary glands, and *R. microplus* BME/CTVM23 cell proteins. Plates were coated with 100 ng proteins per well in carbonate/bicarbonate buffer and incubated overnight at 4 °C. Following five washes with PBS containing 0.05% Tween 20 (PBST), patients and control sera were added at 1:50 dilution in PBS and incubated for 1 h at 37 °C followed by five washes with PBST. For the detection of immunoglobulin, 100 μl of goat anti-human immunoglobulins-peroxidase IgG (FC specific), IgM (μ-chain specific), and IgE (ɛ -chain specific) (Sigma-Aldrich, Madrid, Spain) were added at 1:1000 dilution in blocking buffer (1% HSA in phosphate buffered saline, PBS supplemented with 0.05% Tween 20) (Sigma-Aldrich, Madrid, Spain) (100 μl/well). The plates were then incubated for 1 h at 37 °C and subsequently washed five times with PBST. Reaction was visualized by adding 100 μl of 3,3,´5,5-tetramethylbenzidine (Promega, Madison, WI, USA) and incubated for 20 min in the dark at RT. The optical density (OD) was measured at 450 nm with an ELISA reader. The average value of the blanks (wells without tick proteins coating; *N* = 4) was subtracted from all reads and the average of 4 replicates for each sample was used for analysis.

### Determination of anti-α-Gal IgG, IgM and IgE antibody titers

The anti-α-Gal and total IgE antibody content was determined in patients and control sera using the ImmunoCAP Phadia 250 automated platform (Thermo Fisher Scientific, Uppsala, Sweden) with the commercial kits ImmunoCap α-Gal bovine Thyroglobulin and ImmunoCap Total IgE, respectively, according to the manufacturer's instructions. Levels higher than 100 and 0.35 kU/l were considered positive for total IgE and anti- α-Gal IgE levels, respectively. Anti-α-Gal IgG, IgM and IgE antibody titers were determined in serum samples from patients and the control healthy individual. ELISA plates were coated with 100 μl/well (100 ng) of Gal1-3Galβ1-4GlcNAc-Human serum albumin (HSA) (Carbosynth Ltd, Berkshire, UK) in carbonate/bicarbonate buffer and incubated overnight at 4 °C. Then, 100 μl of blocking buffer (Sigma-Aldrich) were added to each well and incubated 1 hr at RT followed by five washes with PBST. The sera were added to plates at 1:50 dilution in blocking buffer and incubated for 1 hr at 37 °C, followed by five washes with PBST. Goat anti-human immunoglobulins-peroxidase IgG (FC specific), IgM (μ-chain specific), or IgE (ɛ -chain specific) (Sigma-Aldrich) were added at 1:1000 dilution in blocking buffer (100 μl/well), and plates incubated for 1 hr at RT. Plates were then washed five times with PBST, and color developed by the addition of 100 μl of 3,3,´5,5-tetramethylbenzidine (Promega) and protected from the light for 20 min at RT. Reactions were stopped with the addition of 50 μl sulfuric acid, and the OD were measured at 450 nm with an ELISA reader. The average value of the blanks (wells without Galα1-3Galβ1-4GlcNAc-HSA coating; *N* = 4) was subtracted from all reads and the average of 4 replicates for each sample was used for analysis.

### Western blot analyses

1-D Western blot. Twenty μg of *R. bursa* or *H. marginatum* salivary gland and *R. microplus* BME/CTVM23 cell protein extracts were methanol/chloroform precipitated, resuspended in Laemmli sample buffer and separated on a 12% sodium dodecyl sulfate (SDS) polyacrylamide gel under reducing conditions. After electrophoresis, proteins were transferred to nitrocellulose membranes (Bio-Rad, Hercules, CA, USA), blocked with 1% BSA (Sigma) in Tris-buffered saline (TBS; 50 mM Tris-Cl, pH 7.5, 150 mM NaCl) and incubated overnight at 4°C with patient serum (dilution 1:200). To detect the IgE antibodies bound to tick proteins, membranes were incubated with goat anti-human IgE conjugated peroxidase (dilution 1:1000; Sigma). Immunoreactive proteins were visualized with TMB (Promega).

2-D Western blot. Two hundred μg of *H. marginatum* salivary gland or *R. microplus* BME/CTVM23 tick cell proteins were precipitated with chloroform/methanol, resuspended in 500 μl of DeStreak Rehydration Solution™ (GE Healthcare) supplemented with 0.5% of IPG buffer 3-11NL pH range (GE Healthcare) and loaded onto IPG strips (pH 3-11NL Drystrip 7 cm; GE Healthcare). Isoelectrofocusing was performed at 20 °C using an Ettan IPGphor 3 (GE Healtcare) with the following conditions: 30 V for 3 hrs, 60 V for 3 hrs, 60-300 V for 30 min, 300 V for 30 min, 300-1000 V for 1hr, 1000 V for 1hr, 1000-5000 V for 5 hr, 5000 V for 5 hr. Prior to second dimension, proteins present in focused IPG strips were reduced and alkylated by successive incubations in two different equilibration buffer solutions (50 mM Tris-HCl pH 8.8, 6 M urea, 30% v/v glycerol, 2% w/v SDS, 0.2% bromophenol blue, supplemented with either 0.5% w/v dithiothreitol (DTT) for the first incubation or 4.5% w/v iodoacetamide for the second incubation) for 15 min each with gentle rocking. Equilibrated IPG strips were placed onto homogeneous 12% SDS polyacrylamide gels and electrophoresis was carried out for 1h at 120 V. Four 2D gels were performed simultaneously, one was stained with SYPRO^®^ Ruby Protein Gel Stain (Thermo Scientific) and the others were transferred to nitrocellulose membranes to perform Western blots with patients and control sera, following the same protocol described above for 1D Western blot. To detect tick proteins with α-Gal, membranes were blocked with 1% HSA (Sigma) in TBS, incubated overnight at 4°C with anti-α-Gal epitope IgM antibody (Enzo Life Sciences) diluted 1:5 in TBS, and then incubated with anti-mouse IgM conjugated with peroxidase (dilution 1:2000; Sigma). The tick protein spots recognized by patient or control sera and by anti-α-Gal antibodies were manually excised from the stained gel, dehydrated with acetonitrile and vacuum-dried in a speed vacuum centrifuge until proteomics analysis.

### Proteomics analysis

Dried spots were re-hydrated and digested overnight at 37 °C with 12.5 ng/μl trypsin (Promega, Madison, WI, USA) in 50 mM ammonium bicarbonate, pH 8.8 [41]. Trifluoroacetic acid was added to a final concentration of 1 % and the peptides were finally desalted onto OMIX Pipette tips C 18 (Agilent Technologies, Santa Clara, CA, USA), dried-down and stored at -20 °C until used for mass spectrometry (MS) analysis. The desalted protein digests were resuspended in 0.1% formic acid and analyzed by reversed phase (RP) liquid chromatography (LC) coupled to mass spectrometry (RP-LC-MS/MS) using an Easy-nLC II system coupled to an ion trap LTQ-Orbitrap-Velos-Pro mass spectrometer (Thermo Scientific). The peptides were concentrated on-line by reverse phase chromatography using a 0.1 mm x 20 mm C18 RP precolumn (Thermo Scientific), and separated using a 0.075 mm x 100 mm C18 RP column (Thermo Scientific) operating at 0.3 μl/min. Peptides were eluted using a 60 min gradient from 5% to 40% solvent B in solvent A (solvent A: 0.1% formic acid in water, solvent B: 0.1% formic acid, 80% acetonitrile in water). Electrospray ionization (ESI) was carried out using a nano-bore emitters stainless steel ID 30 μm (Thermo Scientific) interface. Peptides were detected in survey scans from 400 to 1600 atomic mass units (amu, 1 μscan), followed by fifteen data-dependent MS/MS scans (Top 15), using an isolation width of 2 mass-to-charge ratio units, normalized collision energy of 35%, and dynamic exclusion applied during 30 sec periods.

Peptide identification from the MS/MS raw data was carried out using the SEQUEST algorithm (Proteome Discoverer 1.4; Thermo Scientific). Database search was performed against the *Rhipicephalus* or *Hyalomma* (12,465 and 5709 entries in November 2015, respectively) Uniprot protein databases. The following constraints were used for the searches: tryptic cleavage after Arg and Lys, up to two missed cleavage sites, and tolerances of 10 ppm for precursor ions and 0.8 Da for MS/MS fragment ions, and the searches were performed allowing optional methionine oxidation and cysteine carbamidomethylation. Searches were performed against a decoy database in an integrated decoy approach. A false discovery rate (FDR) < 0.01 was considered as a condition for successful peptide assignments and at least 2 peptides per protein was the condition for successful protein identification (Supporting information Data Set 1). Functional annotations for identified proteins were assigned using the Blast2GO software (version 3;http://www.blast2go.org/), manually revised and included gene ontology (GO) annotations for biological process, molecular function and cellular component (Supporting information Data Set 1).

### SUPPLEMENTARY MATERIALS DATA SET


